# Characterization of Genome-Wide DNA Methylation and Hydroxymethylation in Mouse Arcuate Nucleus of Hypothalamus During Puberty Process

**DOI:** 10.3389/fgene.2020.626536

**Published:** 2020-12-14

**Authors:** Yihang Shen, Shasha Zhou, Xiaodong Zhao, Hua Li, Jielin Sun

**Affiliations:** ^1^Shanghai Center for Systems Biomedicine, Ministry of Education Key Laboratory for Systems Biomedicine, Shanghai Jiao Tong University, Shanghai, China; ^2^Department of Endocrinology, Shanghai Children's Hospital, Shanghai Jiao Tong University, Shanghai, China; ^3^Bio-ID Center, School of Biomedical Engineering, Shanghai Jiao Tong University, Shanghai, China

**Keywords:** puberty onset, DNA methylation, DNA hydroxymethylation, GnRH, ARC

## Abstract

**Background:** Pulsatile pituitary gonadotropin secretion governed by hypothalamic gonadotropin-releasing hormone (GnRH) is essential for the pubertal onset. The epigenetic mechanism underlying the activation of GnRH-dependent regulatory axis in hypothalamus remains elusive. This study aims to explore the potential correlation between the signature of DNA (hydroxyl)methylation and pubertal process.

**Methods:** Hypothalamic arcuate nucleus (ARC) of mouse at early (4-weeks) and late pubertal (8-weeks) stages underwent RNA-, RRBS-, and RRHP-seq to investigate the genome-wide profiles of transcriptome, differential DNA methylation and hydroxymethylation.

**Results:** A series of differential expressed genes (DEGs) involved in sexual development could be separated into three subgroups with the significant difference of DNA methylation or hydroxymethylation or both in promoter regions. Compared to DNA methylation, DNA hydroxymethylation partook in more signaling pathways including synapse morphology, channel activity and glial development, which could enhance transsynaptic change and glia-to-neuron communication to faciliate GnRH release. The correlation between transcription and these epigenetic modifications indicated that DNA hydroxymethylation impacted with gene transcription independently of DNA methylation spanning puberty.

**Conclusion:** Our results characterized the hydroxymethylation pattern and provided an insight into the novel epigenetic regulation on gene expression during pubertal process.

## Introduction

Pubertal development is a multi-factorial process accompanied by maturity of skeletal height, growth spurt, and a myriad of hormonal changes involving genetic, nutritional, socioeconomic, and environmental factors in a systematic manner leading to reproductive maturation. Pubertal development is governed by the hypothalamic–pituitary–gonadal (HPG) axis, and begins with hypothalamic gonadotropin-releasing hormone (GnRH) neurons. Usually, the dormant HPG axis presents the silencing GnRH, extremely low levels of luteinizing hormone (LH) and follicle stimulating hormone (FSH), and estrogen or testosterone until ~8–9 years of age in human beings (Lee and Houk, 2006, 2008). The pulsatile secretion of GnRH from the hypothalamus stimulates on the gonadotroph cells of the pituitary gland to secrete gonadotropins, LH and FSH, and the gonadotropins then stimulate the production of estrogen from the ovaries in females, and testosterone from the testes in males (Chulani and Gordon, 2014). In turn, the secretion of GnRH in hypothalamus is majorly regulated by KiSS-1 metastasis suppressor (Kiss1) and Kiss1 receptor (Kiss1r, also known as GPR54). Kiss1 neurons of the arcuate nucleus (ARC) in the hypothalamus seem to be essential for pulsatile GnRH release in both sexes. Transcriptional activation of these genes was considered as a core mechanism underlying the puberty initiation, which was precipitated by epigenetic cues (Ojeda and Lomniczi, 2014).

Previous studies have indicated that hypothalamic DNA methylation is strongly implicated in the onset of puberty in mammals (Lomniczi et al., 2015; Yuan et al., 2019). Loss of DNA methylation or demethylation has been observed in specific contexts through active or passive mechanisms (Wu and Zhang, 2010). Active DNA demethylation is the enzymatic process that leads to the removal of the methyl group from 5-methylcytosine (5mC) via successive oxidation [5-hydroxymethylcytosine (5hmC), 5-formylcytosine (5fC), 5-carboxylcytosine (5caC)] catalyzed by ten-eleven translocation (TET) family (Kohli and Zhang, 2013). TET2 has been determined to promote transcription and peptide release of GnRH, and consequently maintain reproductive function in *in vitro* and *in vivo* (Kurian et al., 2016). However, the roles of active DNA demethylation in transcriptional regulation in puberty onset is never elucidated.

Female mice have been widely used in multiple studies on pubertal development as they present the similar molecular behaviors in HPG axis and stable cycles of menstrual calendar like human (Pohl et al., 2007). Hypothalamic ARC underwent a huge epigenetic and genetic reprogramming to adapt to the response and feedback on sexual hormones during the stages of early pubertal (2–5-weeks of age) and late puberty (5–8-weeks of age). Here, we harvested 4- and 8-weeks hypothalamic ARC and employed RNA-seq, reduced representation bisulfite sequencing (RRBS) and hydroxymethylation profiling (RRHP) on a genome-wide scale. Given a large number of differential expressed genes (DEGs) and differential 5(h)mC signals across the whole genome, we discovered novel connections between DNA (hydroxyl)methylated modification and gene expression, emphasizing the importance of epigenetic alterations in regulating transcription during pubertal process.

## Materials and Methods

### Experimental Animals

C57BL/6 female mice purchased from Shanghai SLAC Laboratory Animal Co., Ltd. (Shanghai, China) were housed in clean cages and maintained at 22 ± 2°C with a constant 12-h light/dark schedule. The animals were allowed free access to food and water. 4- and 8-weeks-old mice (*n* = 10 per group) were used in this study. Initially, preliminary experiment for dye injection was used to target the location of ARC using initial orientation (0.4 mm lateral, 1.60 mm posterior to bregma, 7.40 mm below the surface of the dura) as previously described (Greenwood et al., 2014; Hu et al., 2015). Mice were sacrificed via cervical dislocation, and the whole brains were isolated immediately. The hypothalamic ARC tissues in each group were harvested and gathered for the consequent experiments according to the previous dye staining (Supplementary Figure 1A). All the procedures were followed by the Institutional Animal Care and Use Committee of Shanghai Jiao Tong University.

### RNA-seq Library Construction and Data Analysis

ARC tissues were stored in 1 ml TRIZOL (Thermo Fisher Scientific, Waltham, MA, USA) and grinded in liquid nitrogen, and were added 100 μl chloroform and fully mixed, then centrifuged with highest speed at 4°C for 10 min. The supernatant was moved into a new tube, and added the isopropanol with same volume, and centrifuged with highest speed at 4°C for 10 min. The precipitate was washed by 75% cold ethanol, and dissolved by appropriate DEPC water. The concentration and quality of RNA was measured by Nanodrop 2000 (Thermo Fisher Scientific) and Agilent bioanalyzer 2100 (Agilent, Santa Clara, CA, USA). 4 μg of RNA in each group were used for library preparation by NEBNext Ultra Directional RNA Library Prep Kit for Illumina (NEB, Ipswich, MA, USA) following manufacturer's instructions and were sequenced on an Illumina Hiseq platform.

The raw data was trimmed adaptors and filter out low quality reads using Trimmomatic (non-default parameters: SLIDINGWINDOW:4:15 LEADING:10 TRAILING:10 MINLEN:35) (Bolger et al., 2014), and checked the quality of clean reads using Fastqc (Andrews, 2013). Next, clean reads were aligned to the latest mouse genome assembly mm10 using Hisat2 v2.0.5 (non-default parameters: –rna-strandness RF –dta) (Kim et al., 2015). The transcripts were assembled and the expression levels were estimated with FPKM values using the StringTie algorithm (non-default parameters: –rf) (Pertea et al., 2015). Differential mRNA and lncRNA expression among the groups were evaluated using an R package Ballgown (Frazee et al., 2015), and the significance of differences by the Benjamini & Hochberg (BH) *p*-value adjustment method were computed. Gene annotation was described by Ensembl genome browser database (http://www.ensembl.org/index.html). The R package ClusterProfiler was used to annotate the differential genes with gene ontology (GO) terms and Kyoto Encyclopedia of Genes and Genomes (KEGG) pathways (Yu et al., 2012).

### RRBS and RRHP Library Construction and Data Analysis

Genomic DNA of ARC in two groups were extracted using the QIAquick Gel Extraction Kit (Qiagen, Hilden, Germany). The 200 ng high-quality DNA was then digested by restriction endonucleases MspI (NEB) and subjected to 3′-end blunting and single nucleotide (A) addition and adaptor ligation. For RRHP, 5hmC positions at the adapter junctions were modified by T4 phage β-glucosyltransferase (NEB), and non-glucosyl-5hmCs were removed by another round of MspI digestion. The 250–500 bp fragments were then selected and treated with bisulfite conversion using Epitect Bisulfite Kit (Qiagen) according to the manufacturer's instructions. Converted DNA were eluted and performed PCR amplification to enrich for fragments with adapters on both ends. The constructed libraries were quantified using Agilent Bioanalyzer 2100 (Agilent Technologies, Carlsbad, CA, USA) and subjected to high-throughput sequencing using the Illumina Hiseq 2500 platform with paired-end 50 bp sequencing (PE50).

For RRBS, Trim Galore v0.5.0 (non-default parameters: –max-n 0 –length 35 –rrbs) were used to filter adapters, short reads (length < 35 bp) and low quality reads. For RRHP, Cutadapt v1.18 (non-default parameters: –max-n 0 –minimum-length 35), and Trimmomatic v0.38 (non-default parameters: SLIDINGWINDOW:4:15 LEADING:10 TRAILING:10 MINLEN:35) were used to filter adapters, short reads (length < 35 bp) and low quality reads. FastQC (with default parameters) was used to ensure high reads quality. Trimed reads of RRBS data were aligned to reference genome (assembly GRCm38) using Bismark v0.7.0 (with default parameters) and analyzed DNA methylation profiles using methylKit package (Akalin et al., 2012). DMRs were selected by false discovery rate (FDR) < 0.05 and methylation percentage change between control and test groups are > 10%. For RRHP, clean reads were mapped to the mouse genome (assembly GRCm38) using the Bowtie2 v2.3.4.1 (with default parameters) software. Aligned reads with CCGG tag at 5′ end were counted. Differentially hydroxymethylated regions (DHMRs) were determined using the diffReps software. DHMRs were analyzed by log_2_ fold change (FC) >1 or <-1, FDR < 10^−4^.

### Data Deposits

The raw sequencing data was deposited to ArrayExpress assigned with the accession number E-MTAB-9420 and E-MTAB-9421.

## Results

### The Differential Expressed Genes During Pubertal Process

To investigate the changes of epigenome and transcriptome of hypothalamic ARC during puberty progression, we conducted 18 libraries for RNA, RRBS, RRHP-seq derived from hypothalamic ARC of C57BL/6 mice in two sexual developmental stages of early and late puberty. The alignment of data and the correlations within duplications in each group were summarized in Supplementary Table 1 and Supplementary Figure 2, which indicated a good quality of biological materials in this study. As previously described (Li et al., 2017), developmental stages of C57BL/6 mouse were roughly divided into prepuberty and early puberty periods (2–5-weeks of age), late puberty (5–8-weeks of age), and young adulthood (8–12-weeks of age). Here, we detected the vulva morphology and the changes of LH and FSH in sera of 4- and 8-weeks mice, and validated that 8-weeks mice displayed a phenotype of higher hormome levels and gonadal activation compared with 4-weeks mice (Supplementary Figures 1B,C).

**Figure 1:**
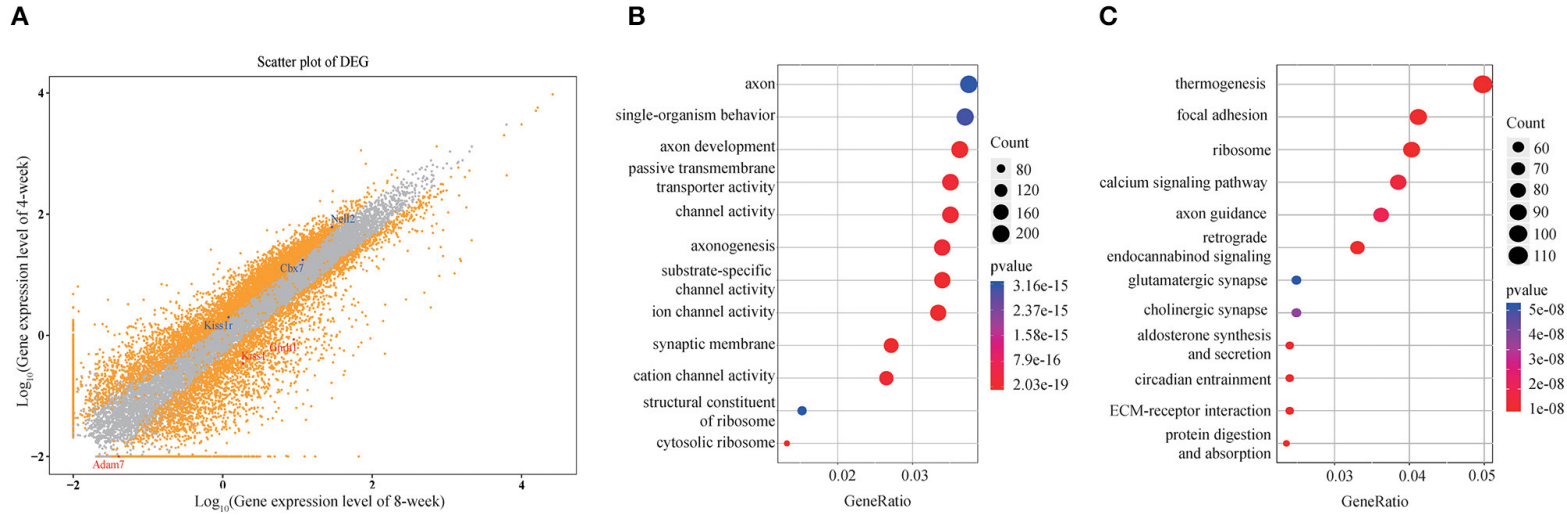
Expression profile of hypothalamic ARC in 4- and 8-weeks. **(A)** Scatter plot of gene expression of 4- /8-weeks. Orange dots represent genes with significant differential expression (*q* < 0.001). Ontology analysis of the involved enriched functions **(B)** and signaling pathways **(C)** of DEGs (*p* < 0.05).

A total of 5,778 DEGs were obtained among which 1,787 protein coding genes were up-regulated while 3,991 were down-regulated in 8-weeks group compared with 4-weeks one (log_2_FC >1 or < −1, FDR <10^−3^). Take an example of the well-acknowledged puberty associated genes, in contrast to the high expression of Kiss1, GnRH, and Adam7 in early pubertal stage of ARC, the presence of substantially decreased Cbx7, Kiss1r, and Nell2 was observed during pubertal process (Figure 1A). Moreover, the functional and signaling pathway enrichment analysis showed that DEGs majorly involved in neurodevelopment, synaptic behavior and transmembrane and extracellular signal transduction (Figures 1B,C), indicating that the specific functional neurons in ARC underwent a complicated process of signals communication and stimulation for maturation. We also observed a high correlation of glutamatergic synapse with puberty (*p* = 5.24 × 10^−8^) and the high expression of glutamate metabolism associated genes such as Grik, Grin and Adcy families in 4-weeks compared with 8-weeks ARC. Previous study indicates that the coordinated activity of glutamatergic neurons and GnRH neurons facilitates the sensitivity of GnRH secretion (Parent et al., 2005). Besides that, choline, aldosterone and endocannabinoid did synthesize and secrete, which likely had profound effects on puberty initiation (Wenger et al., 2002; Biasi, 2010; Genovesi et al., 2018). Taken together, these observations validated the known genetic signatures and indicated multiple activated signaling pathways during pubertal process.

### Genome-Wide Landscape of DNA Methylation and Hydroxymethylation in ARC

**Figure 2:**
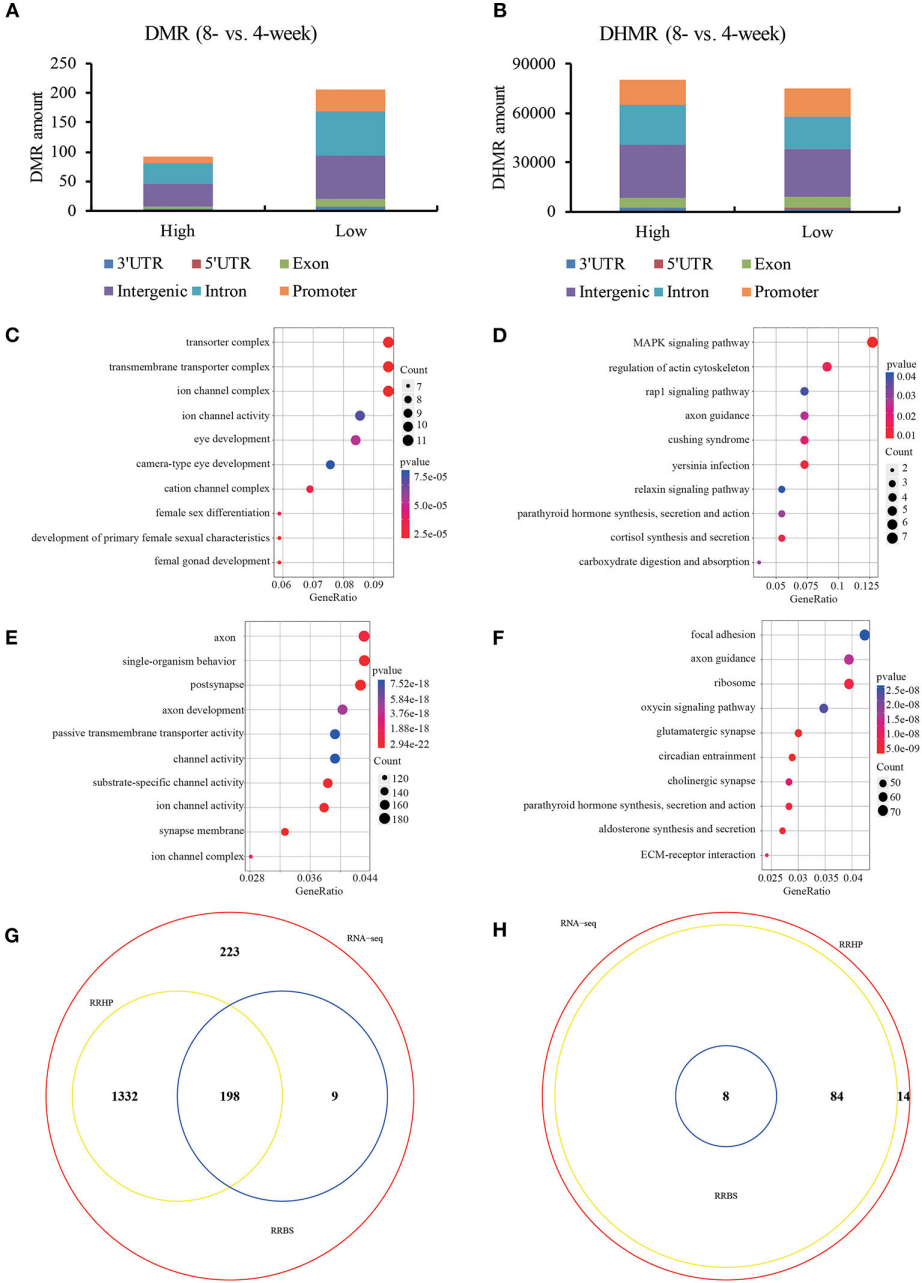
DNA (hydroxy)methylation patterns of hypothalamic ARC in 4- and 8-weeks. The distribution of DMR **(A)** and DHMR **(B)** regions in genomic contexts of DEGs. DMR: differential methylated regions, FC > or < 20%, *q* < 0.05; DHMR: differential hydroxymethylated regions, log2FC >1 or < −1, *q* < 1e-4. Ontology analysis of the involved enriched functions **(C)** and signaling pathways **(D)** of differential methylated DEGs (*p* < 0.05). Ontology analysis of the involved enriched functions **(E)** and signaling pathways **(F)** of differential hydroxymethylated DEGs (*p* < 0.05). The Venn diagram view of enriched functions **(G)** and signaling pathways **(H)** among DEGs with differential (hydroxyl)methylation.

To further investigate the regulatory machinery underlying transcription, RRBS and RRHP were employed to detect the altered genome-wide distribution of 5mC and 5hmC in 4- and 8-weeks old hypothalamic ARC. Our data showed that DMRs and DHMRs (RRBS: FC > or < 10%, FDR < 0.05; RRHP: log2FC >1 or < −1, FDR < 10^−4^) majorly occurred at the promoter, intron and intergenic regions (Figures 2A,B). Here, we focused on the CpG loci located at promoter region of DEGs and observed that these genes whose promoter showed the significantly differential DNA methylation levels between 4- and 8-weeks groups were functionally enriched in signaling pathways closely connected with sexual development and hormone secretion (Figures 2C,D). Likewise, DEGs whose CpG loci at promoter had the remarkable increasing or declining hydroxymethylation levels were majorly associated with the function of synapse morphology and channel activity including glutamatergic synapse (Figures 2E,F). In addition, we found that most of functions and pathways (86.8%) enriched by DHMRs overlapped with those of DEGs compared with DNA methylation (Figures 2G,H). Previous studies have determined that transsynaptic change and glial-neuronal connection attribute to GnRH neuron activation (Ojeda et al., 2010). Current data showed that DHMRs involoved in more genes enriching the functions of glutamate and gamma-aminobutyric acid (GABA) neuron as well as glia development rather than DNA methylation. Our results indicated that DNA methylation might impact with the more intuitive phenotype of hormone secretion while the functions related DNA hydroxymethylation were associated with various auxiliary neurons which were not the most obvious characteristics of sexual development, but were used to trigger and maintain the pubertal initiation. Taken together, the given 5(h)mC patterns in ARC suggested that DNA (hydroxyl)methylation was closely connected with gene expression in puberty onset.

### Independent Roles of DNA Hydroxymethylation and Methylation in Regulating Gene Expression

**Figure 3:**
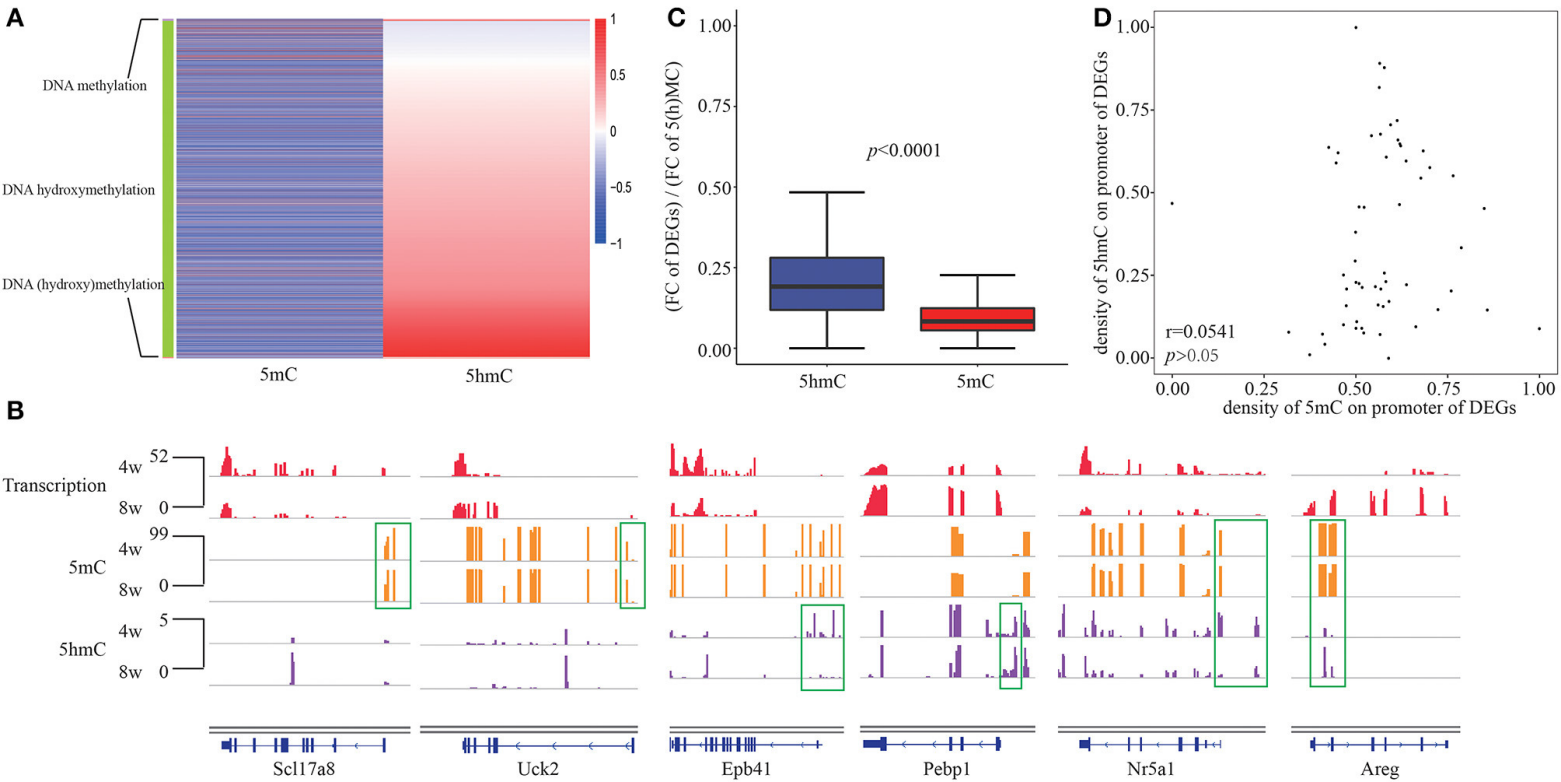
The relationship between DNA hydroxymethylation and transcription. **(A)** Heatmap of 5(h)mC FC at promoter of DEGs between 4- and 8-weeks of hypothalamic ARC. **(B)** Gene browser views of transcription, 5mC and 5hmC profiles in 4- and 8-week of hypothalamic ARC for three clusters of gene regulated by DNA methylation (Scl17a8, Uck2), DNA hydroxymethylation (Epb41, Pebp1), and both (Nr5a1, Areg). **(C)** Comparison of FC of DEGs and D(H)MRs at promoter region. **(D)** The correlation between 5mC and 5hmC of the overlapped D(H)MR in promoter of DEGs.

As well-acknowledgment that DNA methylation in promoters usually negatively correlated with transcription, while DNA hydroxymethylation in promoters displayed a positive correlation with gene expression, we observed the consistent epigenetic regulation of DNA hydroxyl(methylation) on DEGs in our system. Given the overlap of enriched functions between DNA (hydroxy)methylation and DEGs, we grouped three clusters of gene which are negatively correlated with DNA methylation, positively correlated with DNA hydroxymethylation as well as both (Figure 3A). The presence of nine genes including Bhlha15, Insl5, Msmp, Plcb2, Slc17a8, Sox3, Tnfaip2, Uck2, and Ypel2 in first cluster, 3,277 genes including Epb41, Pebp1 in second cluster, as well as 38 genes including Areg and Nr5a1 in third cluster was observed (Figure 3B). During pubertal development, the connection between DNA methylation and hydroxymethylation was seemingly not closed, maybe only in 38 genes of the third cluster.

Moreover, we obtained the FCs of transcription, 5mC and 5hmC between 4- and 8-weeks, and compared the FC of gene expression normalized by the FC of 5(h)mC, indicating that DNA hydroxymethylation could impact gene expression more powerful than DNA methylation (Figure 3C). To further investigate the relationshiop between differential 5mC and 5hmC from the overlapped D(H)MRs, we calculated correlation using Pearson's chi-squared test. Unexpectedly, we failed to observe any significant relativity between each other (*r* = 0.0543, *p* > 0.05) (Figure 3D). Although in consideration of 5hmC as an intermediate of demethylation, however, the results above suggested that DNA hydroxymethylation played a regulatory role in transcription independent of DNA methylation although they had the closely chemical connection.

## Discussion

Although mutations in multiple genes such as kisspeptin system, MKRN3, DLK1 have been identified in sporadic and familial cases of central precocious puberty (CPP), many factors involved in pubertal initiation and transition remain poorly understood (Aguirre and Eugster, 2018). Our data shows that the DEGs of hypothalamic ARC between 4- and 8-weeks include a number of well-acknowledged pubertal associated genes. However, we have meanwhile detected ARC of adult rat, and fail to observe any significant change of Kiss1 and GnRH compared with 4- or 8-weeks (data not shown), which suggests that the expressions of GnRH and other pubertal genes are fluctuant due to the pulsatile release of GnRH and periodic estrus cycle in adult individuals, but their expression changing law during the stages of puberty onset seems more stable than adult stage.

A large number of recent studies have suggested that CpG methylation changes are likely to show a crucial regulatory in controlling the transcription of the well-acknowledged pubertal genes related to GnRH and estrogen signaling pathways in mammalian hypothalamus (Mellen et al., 2012; Alves et al., 2017; Thompson et al., 2018). Although our omics data indicates that DNA methylation change is associated with the expression of a small proportion of genes on gonad development from early to later stages of puberty onset, however, the expressions of more genes are actually impacted by DNA hydroxymethylation at promoter including Kiss1 and Kiss1r. The secretion of GnRH in hypothalamus is majorly regulated by KiSS-1 metastasis suppressor (Kiss1) and Kiss1 receptor (Kiss1r, also known as GPR54). Kiss1 neurons of the arcuate nucleus (ARC) in the hypothalamus seem to be essential for pulsatile GnRH release in both sexes. We speculate that an accompanied genome-wide demethylation processes to facilitate synapse organization to accommodate to regulating the high-level hormone secretion in hypothalamus. 5hmC as a hallmark and an intermediate of demethylation process is determined to play an essential role in normal sexual development in central nervous system in this study. Furthermore, the differential DNA hydroxymethylated genes and their involved functions and pathways have no more than 15% overlap with the ones of DNA methylation, and differences of 5hmC at the promoter of DEGs affect transcription more robustly than 5mC even at the same “CG” loci, which implies that DNA hydroxymethylation exerts an epigenetic regulation independent of DNA methylation although they tightly connect with each other from the chemical basis. 5hmC, which is more than the intermediate of demethylation process *per se*, is likely to reverse the traditional repressive functions of MeCP2 (Mellen et al., 2012), and recruit multiple transcription factors to create an environment to facilitate gene transcription (Ichiyama et al., 2015).

Additionally, we focus on the associated regulatory networks of Kiss1 and GnRH, which seems to be the most important gene for puberty onset. We find that the enriched functions involving Kiss1 but not GnRH from DHMR associated genes and DEGs completely overlapped. We speculated that DNA hydroxymethylation is likely to govern the upstream GnRH regulatory axis in the early stage of puberty initiation. Our data reveals the dynamic DNA (hydroxy)methylation changes of genome of ARC during puberty process, not only indicates the gene expression regulation, but also provides the potential therapeutic targets by epigenetic drugs for puberty associated diseases treatment.

## Conclusion

Overall, our data shows the dynamic change of genome-wide methylation and hydroxymethylation in hypothalamic ARC, and uncovers a novel characterization of DNA hydroxymethylation for regulating transcription during pubertal process. The outcomes advance the understanding on a novel mechanism of epigenetic regulation on gene, and contributes to improving therapeutic strategy for disorders of sex development.

## Data Availability Statement

The raw sequencing data was deposited to ArrayExpress assigned with the accession numbers E-MTAB-9420 and E-MTAB-9421.

## Ethics Statement

The animal study was reviewed and approved by Shanghai Children's Hospital, Shanghai Jiao Tong University.

## Author Contributions

YS analyzed the data, prepared for the figures, and drafted the manuscript. SZ performed the animal experiments and harvested the samples. JS and HL designed the overall project, instructed experiments, and revised the manuscript. XZ helped design the overall project. All authors contributed to the article and approved the submitted version.

## Conflict of Interest

The authors declare that the research was conducted in the absence of any commercial or financial relationships that could be construed as a potential conflict of interest.

## Supplementary Material

Supplementary Figure 1 The phenotype and morphology of pubertal femal mice. **(A)** Dye injection for ARC location. Left: the ventral view of hypothalamus; right: sectional view of coronal suture of hypothalamus. Red pentagon indicates the location of ARC. **(B)** Comparision of vulva morphology between 4- and 8-weeks mice highlighted by the red arrows. The serum levels of LH **(C)** and FSH **(D)** between 4- and 8-weeks mice. “^**^” represent *p* < 0.01.

Supplementary Figure 2 The repeatability and reliability of omics data. Correlation analysis of replicate data, including RNA-seq, RRBS-seq, and RRHP-seq for 4- and 8-weeks ARC samples. Correlations were calculated using whole genome data.
