# Supplementary figures and images for: Characterization of Genome-Wide DNA Methylation and Hydroxymethylation in Mouse Arcuate Nucleus of Hypothalamus During Puberty Process
Source: Front Genet. 2020 Dec 14;11:626536. doi: 10.3389/fgene.2020.626536 (PMC7768033; doi:10.3389/fgene.2020.626536)

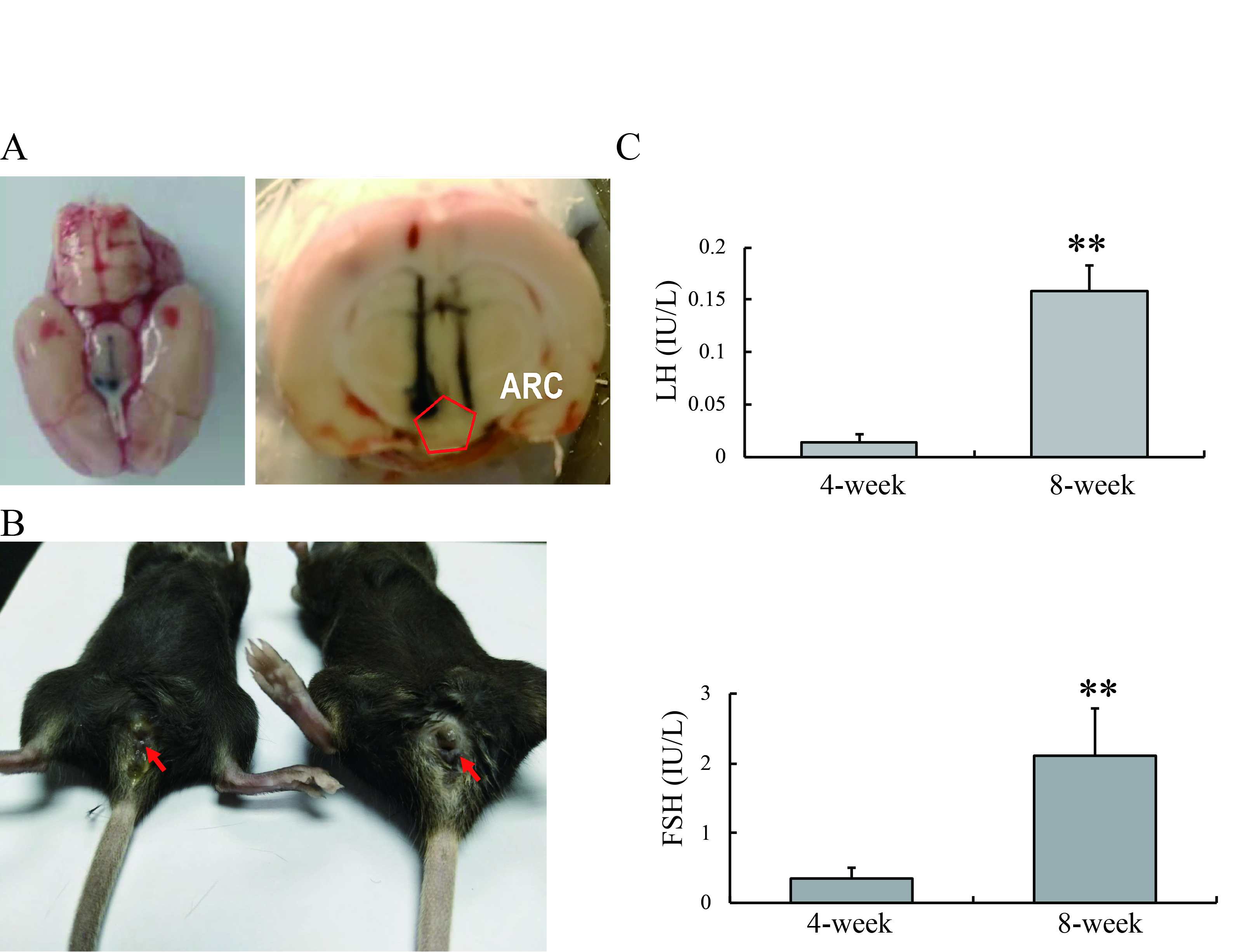

Supplement: Supplementary Figure 1 — The phenotype and morphology of pubertal femal mice. (A) Dye injection for ARC location. Left: the ventral view of hypothalamus; right: sectional view of coronal suture of hypothalamus. Red pentagon indicates the location of ARC. (B) Comparision of vulva morphology between 4- and 8-weeks mice highlighted by the red arrows. The serum levels of LH (C) and FSH (D) between 4- and 8-weeks mice. “**” represent p < 0.01. [file Image_1.TIF]

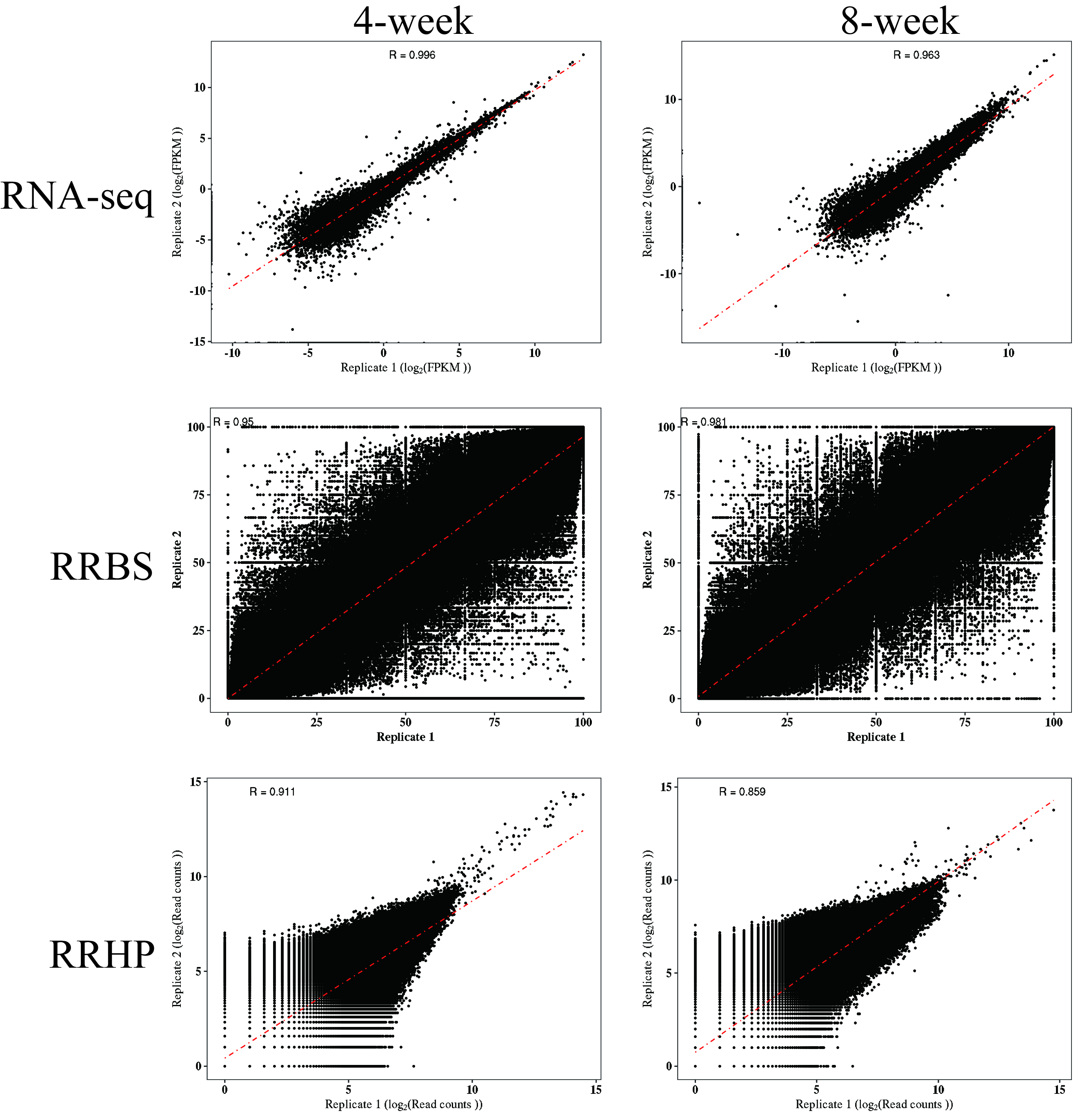

Supplement: Supplementary Figure 2 — The repeatability and reliability of omics data. Correlation analysis of replicate data, including RNA-seq, RRBS-seq, and RRHP-seq for 4- and 8-weeks ARC samples. Correlations were calculated using whole genome data. [file Image_2.TIF]
